# Association between *Helicobacter pylori* infection and the risk of colorectal cancer: A systematic review and meta-analysis

**DOI:** 10.1097/MD.0000000000021832

**Published:** 2020-09-11

**Authors:** Yuling Zuo, Zhao Jing, Mingjiang Bie, Chunyan Xu, Xinyu Hao, Baoning Wang

**Affiliations:** a Hospital of Chengdu University of Traditional Chinese Medicine; b West China fourth hospital of Public Health, Sichuan University; c J. N. Medical Laboratory, Big Data Research Center, University of Electronic Science and Technology of China; d College of Acupuncture-Moxibustion and Tuina, Chengdu University of Traditional Chinese Medicine, Sichuan; e West China School of Basic medical sciences and Forensic Medicine, Sichuan University, Chengdu, China.

**Keywords:** colorectal cancer, *Helicobacter pylori*, heterogeneity, meta-analysis, subgroup analysis, systematic review

## Abstract

**Background:**

The existing evidence on the relationship between *Helicobacter pylori* infection and the risk of colorectal cancer is inconsistent. We conducted a systematic review with a meta-analysis to explore this relationship and to determine whether the relationship varies according to the study characteristics.

**Methods:**

We searched the PubMed, OVID, EMBASE database, and the reference lists of pertinent articles published up to October 2019 by 2 researchers independently. Summary odds ratios (OR) with their 95% confidence intervals (CIs) were estimated using a random-effects model.

**Results:**

Forty seven studies including 17,416 cases of colorectal cancer (CRC) and 55,811 cases of control were included. Overall, *H. pylori* infection was associated with an increased risk of CRC (OR = 1.70 95% CI 1.64–1.76, *I*^2^ = 97%), although there was significant heterogeneity among the studies. Subgroup analysis revealed that the positive correlation might vary by the design of study conducted.

**Conclusion:**

This meta-analysis demonstrates a positive association between *H. pylori* infection and the risk of colorectal cancer.

## Abstract 2

Supplemental Digital Content is available in the text

## 1 Introduction

Colorectal cancer (CRC) is the fourth largest cancer in the world.^[1,2]^ Currently, the cancer burden of CRC is increasing rapidly in China, with an estimate of 1.4 million new CRC cases in 2012, accounting for 9.9% of the global cancer burden. Meanwhile, 693,900 people died, making it the fourth most common cause of cancer-related deaths.^[3]^ In China, the incidence of infection-related cancers are also high^[4]^ accounting for about 17%. Therefore, infection control may also be an effective strategy for cancer prevention.

*Helicobacter pylori* (HP) is a gram-positive pathogen that infects human gastric mucosa and can cause chronic gastritis, peptic ulcer and gastric adenocarcinoma.^[5–7]^ HP infection has become a worldwide problem, with an estimated 4.4 billion people infected with HP in 2015.^[8]^ Since 1994, the international agency for research on cancer has identified HP infection as a major risk factor for gastric cancer.^[9]^ HP infection is well studied in stomach-related diseases, dramatically, persistent inflammation of the stomach caused by HP infection can affect other organs systemically. Especially in recent years, researches on the role of HP in the pathogenesis of extragastric lesions have been widely reported.^[1]^ HP seropositivity in patients has been found to be associated with the increased incidence of various of diseases, including cardiovascular, respiratory, extragastroduodenal, digestive, nervous, and other autoimmune diseases. For example, recent studies have shown that HP may be closely related to cognitive impairment,^[10]^ neurodegenerative diseases,^[11]^ including Parkinson disease,^[12,13]^ Alzheimer disease,^[14]^ depression,^[15]^ the digestive system cancers such as CRC.^[16–20]^ CRC or colorectal adenomas is closely correlated with higher incidence of HP infection in patients.^[18,21,22]^ Previous studies showed inconsistent findings results on the relationship between HP infection and CRC.^[23–27]^ These inconsistent outcomes may be attributed to limited hospital samples from case-control and cross-sectional studies and publish biases from meta-analyses, including poor patient selection and differences in potential confounding factors.^[18,21,22]^ HP-related gastritis is associated with an increased risk of colorectal adenomas and CRC, despite the risk is small.^[28]^ In addition, HP infection was found in the malignant tissues of the CRC.^[29]^ However, the possibility that HP is a direct activator of CRC remains to be a hypothesis. On the other hand, experimental data indicate that a number of potential carcinogenic interactions exist between these bacteria and the colonic mucosa, including the induction and persistence of inflammatory responses, changes in intestinal flora, and the release of toxins and/or hormonal mediators (such as gastrin) that may contribute to tumor formation.^[30,31]^

In present study, we aim to systematically review and summarize the available evidence on the relationship between HP infection and CRC risk, thus assessing the possibility of publication bias, and explore the heterogeneity of the findings.

## 2 Methods and meterials

### 2.1 Data sources and searches

The PRISMA (preferred reporting items for systematic review and meta-analyses) protocol was prospectively conducted.^[32]^ The PubMed, Cochrane databases, and Web of Science databases were searched systematically by 2 researchers respectively from their inceptions to October 2019, using the keywords “*Helicobacter Pylori*”, “*Helicobacter Pylori* infection”, “Colorectal cancer”, “Colorectal cancer”, “Colorectal carcinoma”, “Colorectal tumor” without language restrictions. We also searched the reference lists of all acquired studies to avoid any missing studies. The titles and abstracts were screened firstly by 2 researchers independently. Then, the remaining studies were reviewed by full text and identified based on the inclusion criteria. The disagreement between 2 researchers was solved by discussion. Ethical approval was not necessary, as this study was a meta-analysis based on published studies and did not need handle individual patient data.

### 2.2 Eligibility criteria

Studies that investigated the relationship between HP infection and colorectal cancer were included. Exposure to HP infection was confirmed by invasive tests (endoscopy, biopsy, histopathology), and non-invasive tests (e,^13^C-urea breath test (UBT), immunoglobulin G (IgG) detection, polymerase chain reaction (PCR) and stool antigen); colorectal cancer was confirmed by histological examination. Exclusion criteria as follows:

1. No population studies (e.g., cell lines, animal studies);
2. The literature type is an abstract, letter, review, or other non-research article.

### 2.3 Data extraction

The following information was extracted through predesigned data extraction content by 2 researchers respectively from each included study: publication year, country, authors, sample size, colorectal cancer cases, control cases, HP infection cases, without HP infection cases.

### 2.4 Assessment of quality data synthesis and analysis

We used the Newcastle-Ottawa Scale (NOS) to assess the methodological quality of included studies.^[32]^ The NOS included 3 categories (Selection, Comparability, and Outcome) and 8 items. The NOS ranged from 0 to 9 stars: 4 stars for Selection, 2 stars for Comparability, 3 stars for Outcome. If the total stars was ≥6, we regarded the study as high quality, if the total stars was from 3 to 5, we regarded the study as middle quality; if the total stars was <3, we regarded the study as low quality, and we excluded low quality study. The assessment was conducted by 2 researchers respectively, the disagreement was solved by discussion.

### 2.5 Statistical analysis

The whole manuscript and data were analyzed by RevMan 5.2

(http://ims.cochrane.org/revman/download). The correlation between *H. pylori* infection and colorectal cancer risk was calculated by OR and corresponding 95% confidence interval (95% CI). The heterogeneity across the included studies was assessed by χ^2^ and *I*^2^ tests. The data were pooled by fixed or random effects model according to the heterogeneity test. Random effects model was used if *I*^2^ > 50%. The publication bias was evaluated by Beggs funnel plot. *P* < .05 was considered statistically significant.

## 3 Results

### 3.1 Studies selection and characteristics

**Figure 1:**
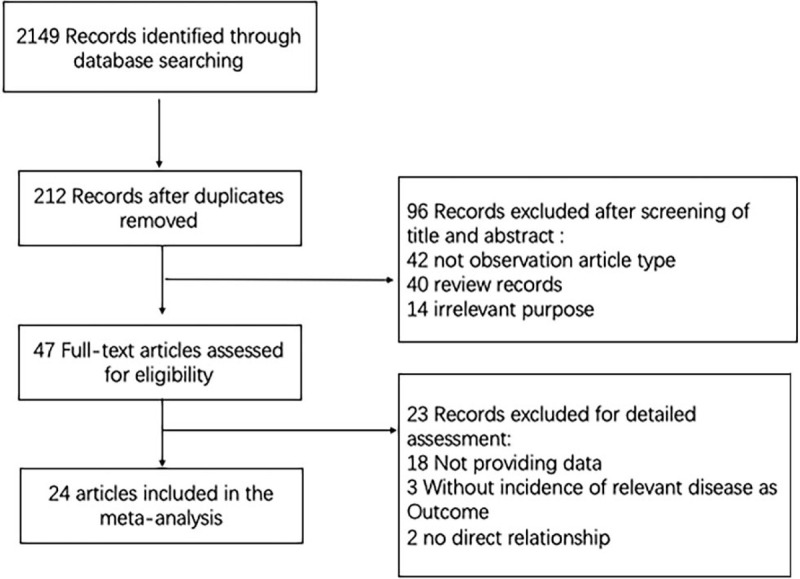
Flow diagram of studies selection.

The detailed study selection progress was shown in Figure 1. Firstly, 2149 studies were identified from PubMed, OVID, and EMBASE. An additional article was included by scanning the reference lists. Finally, 47 studies with 73,227 participants were selected into the meta-analysis.^[17,24,33–47]^ The paper published range from 1991 to 2018 with 17,416 colorectal cancer cases and 55,811 healthy controls. Of these 47 studies, 9 studies were performed in Europe,^[33–41]^ 9 in American,^[17,24,45–50]^ 29 in Asia.^[24,36,42–44,51–60]^ In most studies, the age of participants was above 60, with male accounting for 52%. The characteristics of these studies are summarized in Table 1.

**Table 1:**
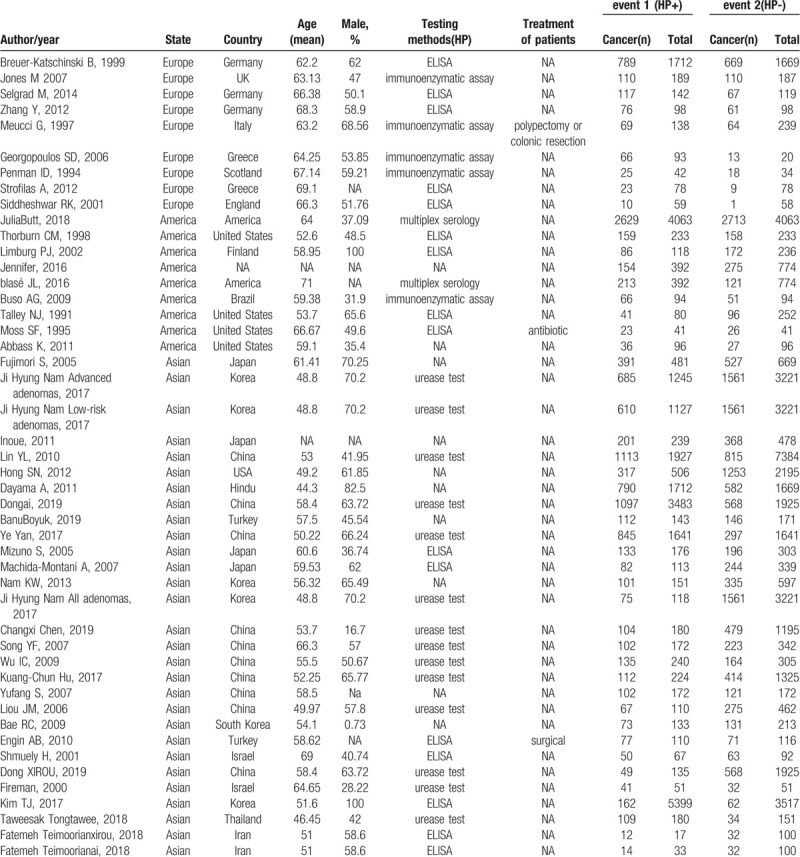
The characteristics of these studies.

Of these 47 studies, 18 studies were high quality,^[24,39,41,46,47,49,50,56,61–62]^ 15 studies were middle quality,^[17,28,33,35,36,38,44,45,56,63–65]^ 14 were low quality.^[34,37,40,42,43,45,48,51,52,54,55,66,67]^

### 3.2 *Helicobacter pylori* and colorectal cancer

**Figure 2:**
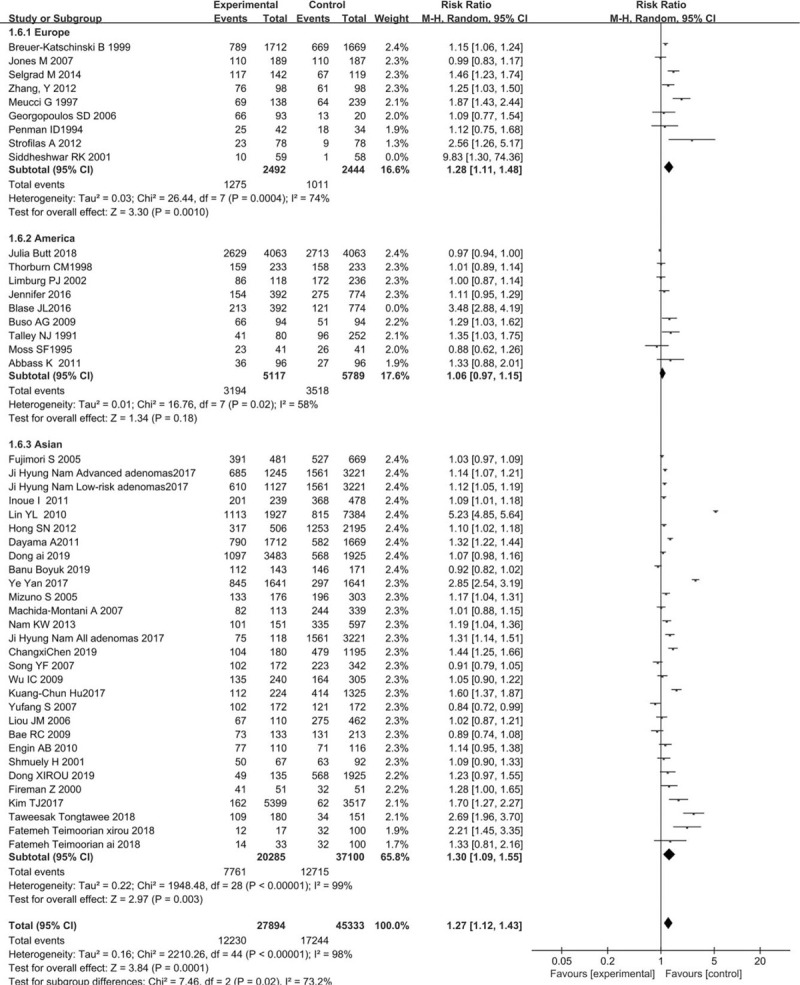
Forest plot of seropositivity to H pylori infection and CRC risk by areas and study. Conditional logistic regression models were applied to determine OR (diamonds) and 95% CI (horizontal lines).

Forty seven case-control studies related to *H. pylori* infection and colorectal cancer risk were eventually included in this meta-analysis. Most studies seemed to show an increase in risk (OR > 1). The results showed that *H. pylori* infection slight increase the risk of developing colorectal carcinoma (OR = 1.70, 95% CI: 1.64–1.76, [Fig. 2].

### 3.3 Subgroup analysis

Subgroup analyses were performed by region of the study conducted. Studies conducted in Asia (OR = 1.98, 95% CI: 1.90–2.07) showed a significantly positive association of risk of CRC and *H. pylori* infection whereas this is not the same in case of studies conducted in the America (OR = 1.14, 95% CI: 1.06–1.23) (Fig. 2).

### 3.4 Publication bias

**Figure 3:**
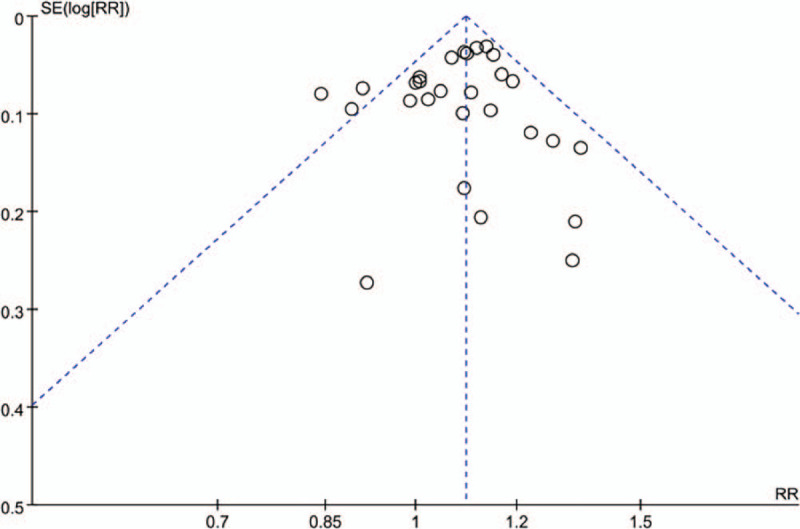
Funnel plot for evaluation the publication bias of *Helicobacter pylori* infection and colorectal cancer risk.

The publication bias for *H. pylori* infection and colorectal cancer risk was evaluated by Beggs funnel plot. The shape of the funnel plots for studies on the association between *H. pylori* infection and the risk of CRC appeared asymmetrical (Fig. 3) and the *P* values for Eggers test (*P* = .03) were indicative of potential publication bias.

### 3.5 Meta-regressions analysis

We conducted 4 meta-regressions based on region, age, country, test-methods respectively, the results are as follows: the Test of Moderators (coefficients 2:4): QM(df = 3) = 1.0395, *P* = .7917, the Test of Moderators (coefficient 2): QM(df = 1) = 0.0261, *P* = .8717, the Test of Moderators (coefficients 2:18): QM(df = 17) = 7.1309, *P* = .9818; the Test of Moderators (coefficients 2:6): QM(df = 5) = 3.0401, *P* = .6938. There is no significant difference (Appendix A, Appendix B, Appendix C, Appendix D).

## 4 Discussion

This meta-analysis analyzed data on HP infection and CRC risk from 2006 to 2019. Interestingly, this study is the first to analyze the relationship between HP infection and CRC risk in large, regionally based populations. All the literatures were analyzed according to subgroups in Europe, Asia, and America. The all literature interestingly, all subgroups exhibited strong heterogeneity including Europe (*I*^2^ = 76%), America (*I*^2^ = 96%), and Asia (*I*^2^ = 98%). The study showed a slight increase in the risk of CRC in all 3 states: American, Europe, and Asia for HP infection.

HP is a recognized class of human carcinogens and has become the main infectious pathogen of single carcinogen in 770,000 cancer cases worldwide every year.^[68]^ Since HP was identified as a single infectious agent for gastric cancer, research into its carcinogenicity has expanded to examine its role in the development of other malignancies.^[69]^ There are conflicting data on the correlation of HP as an etiological factor of CRC. In fact, several studies have reported a slight increase in the risk of CRC associated with HP infection,^[62,68]^ while some reports has demonstrated that HP infection is not associated with CRC risk.^[70]^ This study found a moderate correlation between HP infection and CRC risk. The etiological mechanism of CRC caused by HP has been hypothesized. First, chronic HP infection can lead to hypergastrinemia, which is considered to be a nutrient factor in the colorectal mucosa and may lead to the promoter of mutagenesis. In addition, chronic gastritis caused by helicobacter pylori causes an increase in gastrin production.^[35,71]^ Another possible mechanism is that HP infection causes inflammation, leading to increased production and activity of cyclooxygenase 2 and uraprostaglandin E2, a biomarker associated with inflammation and associated with CRC risk.^[72]^ Finally, recent studies have shown that certain components of HP cell wall have carcinogenic effects on the colorectal epithelial cells. Taken together, these data support the etiological role of HP in the development of colorectal tumors.

It is well known that HP infection rates are different in the general population of western and eastern countries.^[73]^ Therefore, we performed subgroup analyses for Asia, Europe, and the American based on regional differences in populations. The results showed that positive correlation was found in the HP infection and the risk of CRC in different subgroup populations, including Asian, American, and European populations. The strength of this study is, firstly, we performed multi-subgroup analysis to test the robustness of our results in the selection of study methods and other confounding factors.

Since meta-analysis belongs to observational study in nature, we should be particularly careful in interpreting the analysis results, mainly considering homogeneity and its impact on the results. The analysis results should not be separated from the professional knowledge background and should have practical significance. In etiological case-control studies, odds ratio (OR) is the most often used value to estimate the strength of the association between exposure factors and disease, and meta-analysis should also be performed on multiple studies with the same purpose to comprehensively and quantitatively evaluate the strength of the association between exposure and disease. In this study, our findings reported that risk of CRC the in all populations were correlated with HP infection with an OR = 1.7, CI (1.64–1.76). In addition, for subgroup analysis, risk of CRC the in the following populations were separately American (OR = 1.08, CI (0.9–1.3)), Europe (OR = 1.28, CI (1.11–1.48)) and Asia (OR = 1.3, CI (1.09–1.55)) for HP infection, which suggest that HP infection is associated with the risk of CRC in Asia, American and Europe. However, we cannot explain all confounding variables in this study. In meta-regression and subgroup analyses, firstly, we found that the risk assessment for CRC associated with HP infection was robust and stable across a variety of study characteristics. Secondly, the results of the funnel plots migrations and Eggers tests excluded the possibility of publication bias. Studies with statistically significant results are more likely to be published than those with no significant results. Thirdly, limited information about the anti-HP treatment were recorded in patients. Whether treatment with eradication therapy for HP-infected subjects reduces the risk of CRC is an open question.

One limitation of this study is that potential information variables such as recent antibiotic use, gastritis diagnosis and inflammatory bowel disease were excluded from most of the subjects. These variables may increase information about active HP infection and/or eradication of HP infection at the time of blood drawing, as well as potential links between HP infection and inflammatory bowel disease. In this context, it is important to note that serological analysis of HP infection as a systematic measure of past and/or acute infection does not provide information on an individuals current site-specific infection status. In addition, there was still a large amount of missing data about potential confounding factors, such as CRC testing for family history that we were not able to control for effectively.

## 5 Conclusion

In summary, this meta-analysis found a modest association between HP infection and CRC risk in Asian, America, and European populations. However, more prospective, high-quality controlled studies are needed to confirm our findings.

## Author contributions

**Methodology:** Xinyu Hao.

**Writing – review & editing:** Xinyu Hao.

## Supplementary Material

Supplemental Digital Content

## Supplementary Material

Supplemental Digital Content

## Supplementary Material

Supplemental Digital Content

## Supplementary Material

Supplemental Digital Content
