# Supplementary material for: Association between Helicobacter pylori infection and the risk of colorectal cancer: A systematic review and meta-analysis
Source: Medicine (Baltimore). 2020 Sep 11;99(37):e21832. doi: 10.1097/MD.0000000000021832 (PMC7489651; doi:10.1097/MD.0000000000021832)

Study or Subgroup	Experimental Events	Experimental Total	Control Events	Control Total	Weight (fixed)	Weight (random)	Odds Ratio MH, Fixed + Random, 95% CI
region = Europe							
SiddheshwarRK, 2001	10	59	1	58	0.0%	0.8%	11.63 [1.44, 94.12]
Breuer-KatschinskiB, 1999	789	1712	669	1669	7.9%	2.4%	1.28 [1.11, 1.46]
SelgradM, 2014	117	142	67	119	0.3%	2.1%	3.63 [2.07, 6.38]
Zhang.Y, 2012	76	98	61	98	0.3%	2.0%	2.10 [1.12, 3.92]
StrofilasA, 2012	23	78	9	78	0.1%	1.8%	3.21 [1.37, 7.49]
GeorgopoulosSD, 2006	66	93	13	20	0.1%	1.7%	1.32 [0.47, 3.66]
MeucciG, 1997	69	138	64	239	0.5%	2.2%	2.73 [1.76, 4.24]
PenmanID, 1994	25	42	18	34	0.2%	1.8%	1.31 [0.52, 3.26]
JonesM, 2007	110	189	110	187	1.0%	2.2%	0.97 [0.65, 1.47]
Total (fixed effect, 95% CI)		2551		2502	10.4%	--	1.45 [1.29, 1.63]
Total (random effects, 95% CI)					--	17.0%	1.93 [1.33, 2.79]
Heterogeneity: $Tau^2 = 0.19$; $Chi^2 = 33.67$, df = 8 ($P < 0.01$); $I^2 = 76\%$							
Test for overall effect (fixed effect): $Z = 6.33$ ($P < 0.01$)							
Test for overall effect (random effects): $Z = 3.48$ ($P < 0.01$)							
region = America							
JuliaButt, 2018	2629	4063	2713	4063	20.6%	2.4%	0.91 [0.83, 1.00]
Jennifer, 2016	154	392	275	774	2.4%	2.3%	1.17 [0.91, 1.51]
AbbassK, 2011	36	96	27	96	0.4%	2.1%	1.53 [0.84, 2.81]
Total (fixed effect, 95% CI)		4551		4933	23.4%	--	0.95 [0.87, 1.03]
Total (random effects, 95% CI)					--	6.7%	1.07 [0.83, 1.38]
Heterogeneity: $Tau^2 = 0.03$; $Chi^2 = 5.88$, df = 2 ($P = 0.05$); $I^2 = 66\%$							
Test for overall effect (fixed effect): $Z = -1.20$ ($P = 0.23$)							
Test for overall effect (random effects): $Z = 0.52$ ($P = 0.60$)							
region = America							
BlaseJL, 2016	213	392	121	774	0.8%	2.3%	6.42 [4.86, 8.48]
BusoAG, 2009	66	94	51	94	0.3%	2.1%	1.99 [1.09, 3.62]
LimburgPJ, 2002	86	118	172	236	0.7%	2.2%	1.00 [0.61, 1.64]
ThorburnCM, 1998	159	233	158	233	1.1%	2.2%	1.02 [0.69, 1.51]
TalleyNJ, 1991	41	80	96	252	0.5%	2.1%	1.71 [1.03, 2.84]
MossSF, 1995	23	41	26	41	0.2%	1.8%	0.74 [0.30, 1.79]
Total (fixed effect, 95% CI)		958		1630	3.6%	--	2.37 [2.00, 2.82]
Total (random effects, 95% CI)					--	12.7%	1.63 [0.74, 3.62]
Heterogeneity: $Tau^2 = 0.91$; $Chi^2 = 87.68$, df = 5 ($P < 0.01$); $I^2 = 94\%$							
Test for overall effect (fixed effect): $Z = 9.83$ ($P < 0.01$)							
Test for overall effect (random effects): $Z = 1.21$ ($P = 0.23$)							
region = Asian							
ShmuelyH, 2001	50	67	63	92	0.3%	2.0%	1.35 [0.67, 2.74]
FujimoriS, 2005	391	481	527	669	1.8%	2.3%	1.17 [0.87, 1.57]
Dongai, 2019	1097	3483	568	1925	10.8%	2.4%	1.10 [0.97, 1.24]
YeYan, 2017	845	1641	297	1641	3.1%	2.4%	4.80 [4.10, 5.63]
ChangxiChen, 2019	104	180	479	1195	1.1%	2.3%	2.05 [1.49, 2.81]
SongYF, 2007	102	172	223	342	1.3%	2.2%	0.78 [0.53, 1.13]
WuIC, 2009	135	240	164	305	1.4%	2.3%	1.11 [0.79, 1.55]
LiouJM, 2006	67	110	275	462	0.9%	2.2%	1.06 [0.69, 1.62]
DongXIROU, 2019	49	135	568	1925	1.0%	2.3%	1.36 [0.95, 1.96]
YufangS, 2007	102	172	121	172	1.1%	2.2%	0.61 [0.39, 0.96]
LinYL, 2010	1113	1927	815	7384	3.1%	2.4%	11.02 [9.81, 12.38]
Kuang-ChunHu, 2017	112	224	414	1325	1.3%	2.3%	2.20 [1.65, 2.93]
DayamaA, 2011	790	1712	582	1669	6.8%	2.4%	1.60 [1.39, 1.84]
FatemehTeimoorianxirou, 2018	12	17	32	100	0.1%	1.6%	5.10 [1.66, 15.70]
FatemehTeimoorianai, 2018	14	33	32	100	0.2%	1.9%	1.57 [0.70, 3.51]
Fireman, 2000	41	51	32	51	0.1%	1.8%	2.43 [1.00, 5.95]
MizunoS, 2005	133	176	196	303	0.8%	2.2%	1.69 [1.11, 2.56]
Machida-MontaniA, 2007	82	113	244	339	0.7%	2.2%	1.03 [0.64, 1.66]
Inoue, 2011	201	239	368	478	0.8%	2.2%	1.58 [1.05, 2.38]
Nam JH, 2017	685	1245	1561	3221	8.4%	2.4%	1.30 [1.14, 1.48]
Nam JH, 2017	610	1127	1561	3221	8.0%	2.4%	1.25 [1.10, 1.44]
Nam JH, 2017	75	118	1561	3221	0.9%	2.2%	1.85 [1.27, 2.72]
KimTJ, 2017	162	5399	62	3517	1.6%	2.3%	1.72 [1.28, 2.32]
NamKW, 2013	101	151	335	597	1.0%	2.2%	1.58 [1.09, 2.30]
BaeRC, 2009	73	133	131	213	1.0%	2.2%	0.76 [0.49, 1.18]
TaweesakT, 2018	109	180	34	151	0.3%	2.2%	5.28 [3.25, 8.58]
EnginAB, 2010	77	110	71	116	0.4%	2.1%	1.48 [0.85, 2.57]
BanuBoyuk, 2019	112	143	146	171	0.6%	2.1%	0.62 [0.35, 1.11]
HongSN, 2012	317	506	1253	2195	3.8%	2.3%	1.26 [1.03, 1.54]
Total (fixed effect, 95% CI)		20285		37100	62.6%	--	1.98 [1.90, 2.07]
Total (random effects, 95% CI)					--	63.6%	1.60 [1.16, 2.21]
Heterogeneity: $Tau^2 = 0.73$; $Chi^2 = 1314.93$, df = 28 ($P < 0.01$); $I^2 = 98\%$							
Test for overall effect (fixed effect): $Z = 30.77$ ($P < 0.01$)							
Test for overall effect (random effects): $Z = 2.86$ ($P < 0.01$)							
**Total (fixed effect, 95% CI)**		**28345**		**46165**	**100.0%**	**--**	**1.70 [1.64, 1.76]**
**Total (random effects, 95% CI)**					**--**	**100.0%**	**1.64 [1.29, 2.08]**

Heterogeneity: $Tau^2 = 0.62$; $Chi^2 = 1700.11$, df = 46 ($P = 0$); $I^2 = 97\%$
Residual heterogeneity: $Tau^2 = NA$; $Chi^2 = 1442.17$, df = 43 ($P < 0.01$); $I^2 = 97\%$
Test for overall effect (fixed effect): $Z = 29.09$ ($P < 0.01$)
Test for overall effect (random effects): $Z = 4.05$ ($P < 0.01$)
Test for subgroup differences (fixed effect): $Chi^2 = 248.97$, df = 3 ($P < 0.01$)
Test for subgroup differences (random effects): $Chi^2 = 7.99$, df = 3 ($P = 0.05$)

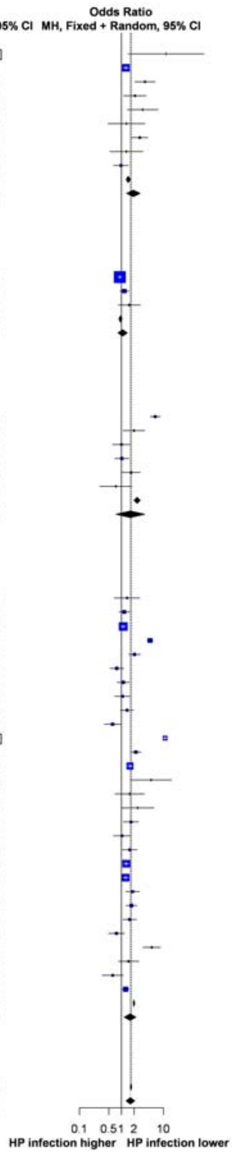

Supplement: Supplemental Digital Content [file medi-99-e21832-s001.pdf]
