# Supplementary figures and images for: Association between Helicobacter pylori infection and the risk of colorectal cancer: A systematic review and meta-analysis
Source: Medicine (Baltimore). 2020 Sep 11;99(37):e21832. doi: 10.1097/MD.0000000000021832 (PMC7489651; doi:10.1097/MD.0000000000021832)

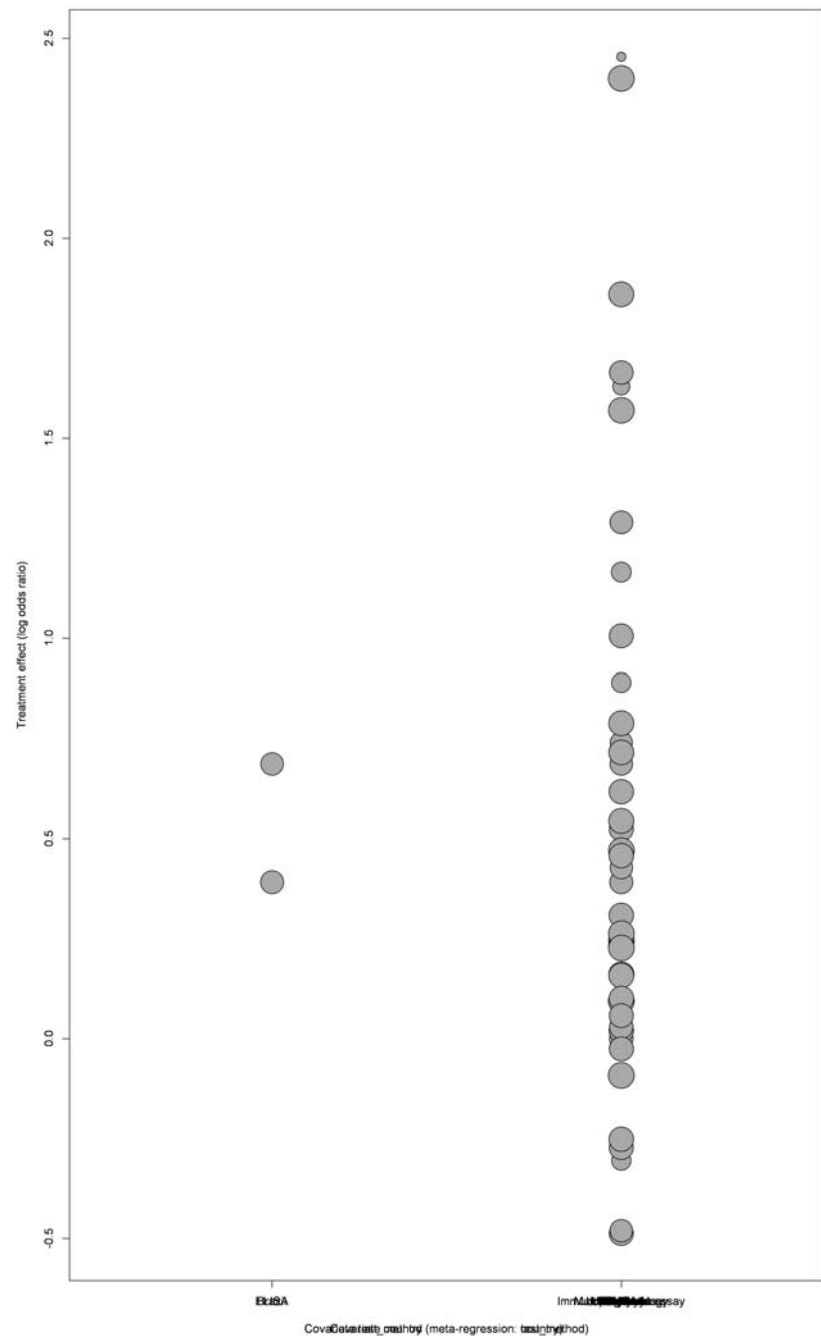

Supplement: Supplemental Digital Content [file medi-99-e21832-s002.pdf]

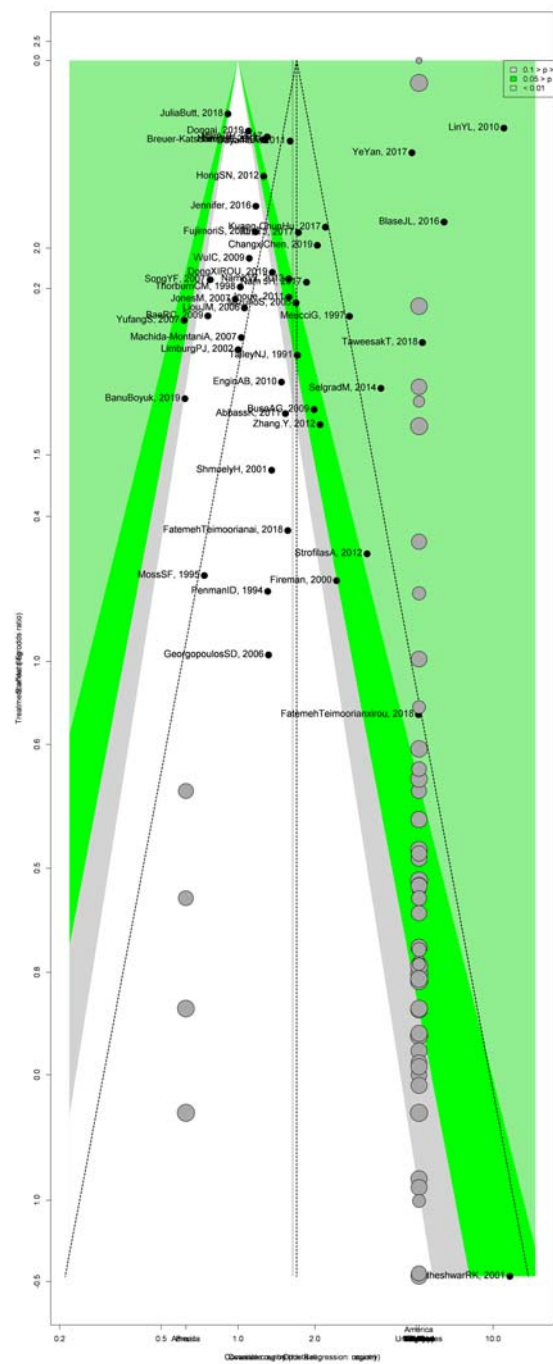

Supplement: Supplemental Digital Content [file medi-99-e21832-s003.pdf]
